# Assessment of heterogeneous Head Start treatment effects on cognitive and social-emotional outcomes

**DOI:** 10.1038/s41598-022-10192-1

**Published:** 2022-04-19

**Authors:** Sun Yeop Lee, Rockli Kim, Justin Rodgers, S. V. Subramanian

**Affiliations:** 1 Department of Epidemiology, Harvard T.H. Chan School of Public Health, Boston, MA USA; 2 Division of Health Policy and Management, College of Health Sciences, Korea University, Seoul, South Korea; 3 Interdisciplinary Program in Precision Public Health, Department of Public Health Sciences, Graduate School of Korea University, Seoul, South Korea; 4 Harvard Center for Population and Development Studies, Cambridge, MA USA; 5 Department of Social and Behavioral Sciences, Harvard T.H. Chan School of Public Health, Boston, MA USA

**Keywords:** Risk factors, Paediatric research

## Abstract

Head Start is a federally funded, nation-wide program in the U.S. for enhancing school readiness of children aged 3–5 from low-income families. Understanding heterogeneity in treatment effects (HTE) is an important task when evaluating programs, but most attempts to explore HTE in Head Start have been limited to subgroup analyses that rely on average treatment effects by subgroups. This study applies an extension of multilevel modelling, complex variance modelling, to data from a randomized controlled trial of Head Start, Head Start Impact Study (HSIS). The treatment effects on the variance, in addition to the mean, of nine cognitive and social-emotional outcomes were assessed for 4,442 children aged 3–4 years who were followed until their 3rd grade year. Head Start had positive short-term effects on the means of multiple cognitive outcomes while having no effect on the means of social-emotional outcomes. Head Start reduced the variances of multiple cognitive and one social-emotional outcomes, meaning that substantial HTE exists. In particular, the increased mean and decreased variance reflect the ability of Head Start to improve the outcomes and reduce their variability. Exploratory secondary analyses suggested that larger benefits for children with Spanish as a primary language and low parental educational level partly explained the reduced variability, but the HTE remained and the variability was reduced even within these subgroups. Routinely monitoring the treatment effects on the variance, in addition to the mean, would lead to a more comprehensive program evaluation that describes how a program performs on average and on the entire distribution.

## Introduction

Program evaluations generally focus on assessing average treatment effect (ATE) which is estimated by the difference in an outcome variable between those who are treated versus not treated. However, reporting of ATE as a single number summary of all individual treatment effects can be misleading as it dismisses the heterogeneity around the group average.^1,2^ If the heterogeneity in the treatment effects (HTE) were meaningfully large, the ATE would be insufficient in describing how well and for whom the intervention worked.^3,4^ Policies and interventions guided by such an ATE estimate would be ineffective, especially when deciding to scale up the intervention, as they would not meet heterogeneous needs of individuals.

Head Start is one example of a governmental program scaled up without an adequate understanding of its HTE. Initiated in 1965 in the U.S., the federally funded child developmental program aims to enhance school readiness of children aged 3–5 from low-income families by providing educational, health, nutritional, and social services.^5^ Across the country, it has served more than 37 million children and their families since. Head Start and its expanded version to infants and toddlers, Early Head Start, have been successful in receiving bipartisan support and saw an $890 million increase in funding between fiscal year 2016 and 2019, and its funding was set at $10.61 billion in 2020. Understanding HTE is especially important for such a nation-wide program with many recipients. In 2002, the Head Start Impact Study (HSIS), a nationally representative randomized controlled trial (RCT), was launched to evaluate the effectiveness of Head Start. Official reports of the HSIS documented positive short-term effects on some cognitive, social-emotional, health, and parenting outcomes, but null long-term effects for most outcomes.^6,7^ Recognizing that child development interventions like Head Start may have meaningfully large HTE,^8–10^ subsequent studies have moved beyond the assessment of ATE. They further examined for which subgroups of children Head Start was effective and found that the program in general had compensatory effects, or greater benefits for those with greater needs.^11^ Head Start benefitted several subgroups with more disadvantages, such as children with Spanish as a primary language,^12,13^ those who had lower cognitive skills at baseline,^12,13^ and those with home-based or non-parental care.^14–16^ However, further examination of HTE in Head Start is needed because findings on the treatment effects were mixed for many other disadvantaged subgroups, such as children with low parental education level,^17,18^ special needs,^19^ single parents,^20^ or caregivers with depressive symptoms.^21^.

A common methodological approach of the previous studies on HTE was a subgroup analysis which restricts the analysis to a subgroup or tests for statistical interactions between the treatment and covariates of interest (e.g., gender, race/ethnicity).^11^ However, such an approach has been shown to be insufficient in capturing HTE because it still relies on ATE.^22^ While one can test whether ATE estimates are heterogeneous across selected subgroups, heterogeneity around those estimates remains masked. Indeed, using the HSIS data, Ding, Feller, & Miratrix^23,24^ found substantial HTE beyond what the observed covariates and treatment noncompliance can explain, suggesting that different approaches are necessary to better understand HTE in Head Start.

Complex variance modelling, an extension of multilevel modelling, is one way to examine HTE.^25–28^ Instead of making the common assumption of constant variance (i.e., homoscedasticity), it explicitly models the variance, in addition to the mean, of an outcome as a function of covariates. By analyzing the treatment effect on the entire outcome distribution, individual variability is a main estimand of interest without being sidelined by the simple average. At baseline of a well-designed RCT with sufficient sample size, the variance, as well as the mean, of an outcome is expected to be comparable across treatment and control groups. In turn, a substantial difference in post-treatment variance between the two groups would be a systematic phenomenon and could be attributed to HTE.^1^ Such treatment effect on the variance would indicate that the ATE estimate alone does not sufficiently describe for whom the treatment worked and warrant further investigation.

Another important information that complex variance modelling provides is the magnitude and direction of the effect on variance. In many cases, societal-level governmental programs and policies aim to not only improve an outcome on the average, but also reduce social inequality in the outcome.^29,30^ Head Start, for example, helps low-income children, who generally score lower on school readiness than the country average, to ultimately pull them up towards the mean, reducing the variance in addition to increasing the overall mean. Even among low-income children that are targeted by the program, the academic performance gap can exist, and under the spirit of Head Start, it would be ideal to benefit every child but more for those at the lower part of the outcome distribution, thereby increasing the mean and reducing the variance. In such a way, when statistical analyses consider the mean and variance simultaneously, the treatment effect can be described in nine possible scenarios.^1^ The mean can increase, decrease, or be left unchanged, and for each of these cases, the variance can increase, decrease, or be left unchanged. For example, the increased (improved, in this case) mean with the decreased variance could mean that those who were lower at the outcome distribution were able to benefit from the intervention and perhaps for those at the higher tail of the distribution, to a lesser degree or not at all. No change for the mean with the increased variance may mean some were benefitted, some were harmed, or both. If the treatment effect is evaluated in these nine scenarios, our understanding of the impact of an intervention would be more comprehensive.

One study has applied complex variance modelling on the HSIS data and found reduced variance of cognitive outcomes among the Head Start children, but the effect on the variance was not interpreted with the effect on the mean.^13^ Other methodological approaches to the distributional effect, such as quantile regressions, were also implemented and found that Head Start benefitted those at the lower tail of the outcome distribution more for cognitive outcomes.^12,14^ However, both of these studies analyzed only the small number of outcomes at the 1^st^ follow-up year of the 6-year-long study.

Using the HSIS data, the present study applied complex variance modelling on nine child developmental outcomes (six cognitive and three social-emotional outcomes) at four time points (1st, 2nd, 3rd, and the 3rd grade follow-up years) to comprehensively analyze the effect of Head Start. We visualized the treatment effect on the entire outcome distribution and interpreted the treatment effect based on the ATE (i.e., the effect on the mean) and the individual variability (i.e., the effect on the variance). Then, to further investigate HTE, we conducted exploratory subgroup analyses with complex variance modelling. The subgroups were specified post-hoc and based on a primary language (English or Spanish) and a parental education level (high school graduates, less, or more).

## Methods

### Sample

**Figure 1:**
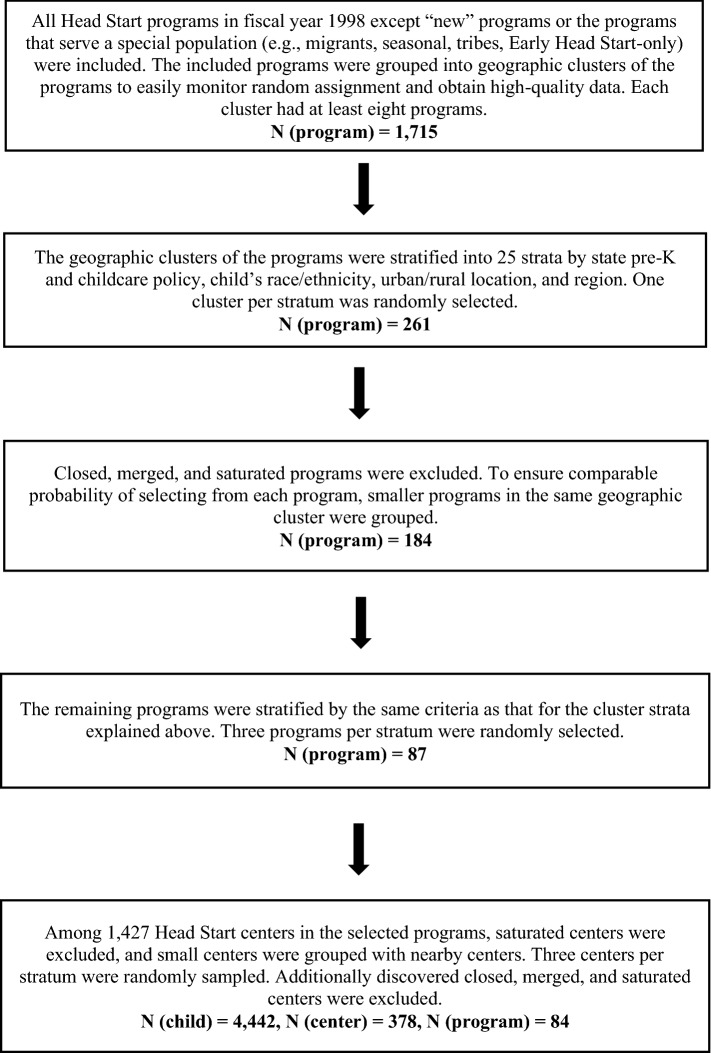
Multi-stage sampling process for a nationally representative Head Start children.

The HSIS utilized multi-stage sampling to select Head Start programs, centers, and participants (Fig. 1).^6,7^ First, all Head Start programs that operated less than two years, those that only served a special population (e.g., migrant, seasonal, tribal), and Early Head Start programs were excluded. The remaining 1,715 programs were grouped into 161 geographic clusters to easily monitor random assignment and obtain high-quality data. After stratifying the clusters by contextual criteria (i.e., state pre-K and childcare policy, child race/ethnicity, urban/rural location, and region), one cluster per stratum was randomly selected, resulting in 261 programs. Programs that were closed, merged, or saturated (i.e., being able to serve all applicants)were excluded. Only programs that had more applicants than available spots (i.e., not saturated) were included so that a control group could be formed. Small programs in the same geographic cluster were grouped to ensure a comparable probability of being selected across programs, resulting in 184 programs. These programs were once again stratified by the contextual criteria considered above to create strata, and three programs were randomly selected per cluster. In the selected 87 programs, there were 1,427 centers potentially eligible for the study. These centers were stratified into strata based on the same contextual criteria, and three centers per stratum were randomly sampled. All Head Start applicant children in the selected centers were included in the final sample, which consisted of 4,442 children in 378 centers out of 84 programs. Additional details are available in the HSIS official reports^6,7^.

### Treatment

The Head Start intervention included educational, health, nutritional, and social services with the goal of improving school readiness and child development. All Head Start centers must adhere to the Head Start Performance Standards, which are federally regulated to ensure the comprehensiveness and quality of the services provided by the centers.^6^ Thus, the treatment is a mixture of various services with the pre-specified standards. With such multidimensional treatment, a precise mechanism through which Head Start affects children is challenging to uncover. Nonetheless, the overall impact of the national-level program and its heterogeneity can be evaluated.

Randomization of Head Start occurred within each Head Start center in the first year of the HSIS. The treatment group (or the Head Start children) were offered to participate in Head Start, while the control group (or the Control children) were not. The randomization was designed to yield a higher proportion of children having access to Head Start in order to allow as many children as possible to be potentially benefitted from the program.

For both the 3- and 4-year-old cohorts, the treatment of interest is the offer of one year of Head Start. Unlike the 4-year-old cohort who had only one eligible year for Head Start (i.e., the first year, or the randomization year), the 3-year-old cohort had one more eligible year (i.e., the second year, or the year after the randomization year) when they turned age 4. However, for that year, both the Head Start children and the Control children were free to enroll in Head Start. It was not reasonable to prevent 3-year-old children from enrolling in Head Start for two years. Therefore, the treatment is the same for both cohorts in that it offers one year of Head Start. One important difference is that the 3-year-old cohort has an opportunity to enroll again in the next year, while the 4-year-old cohort does not have an opportunity to enroll again.

The Control children were prevented from enrolling in the Head Start center where they applied, but their alternative experiences were not controlled. Therefore, their experiences range widely from non-Head Start childcare programs to home care. About 60% of the Control children participated in non-Head Start childcare programs. In addition, as with any RCT, there was noncompliance to the random assignment; 12% of the Control children enrolled in Head Start, and 19% of the Head Start children did not actually enroll in Head Start. In summary, the causal question of this RCT is whether one year of Head Start had an impact on children’s developmental outcomes when compared against a mixture of alternative experiences that low-income children would have had if Head Start did not exist.

### Outcomes

The participating children were followed up and assessed for multiple cognitive and social-emotional outcomes at preschool years, kindergarten year, the 1^st^ grade year, and the 3^rd^ grade year. Since all outcomes measured in the HSIS have theoretical reasons to believe that they may be influenced by Head Start, we would ideally analyze as many outcomes as possible so that we can identify unexplored HTE to better understand the effects of Head Start and demonstrate the utility of complex variance modelling. However, based on the following criteria, only outcomes with reliable data quality and that are compatible with our analytical approach are selected. Outcomes were excluded if there were: (1) no or limited evidence on reliability of the measure, (2) problems raised in the HSIS official reports on scoring and interpreting results, (3) subjective academic performance measures in the presence of comparable objective measures, (4) measures not available for both 3- and 4- year-old cohorts at a given follow-up assessment, and( 5) in a categorical form. The final outcome selections were six cognitive outcomes (Peabody Picture Vocabulary Test (PPVT),^31^ Woodcock-Johnson (WJ) III Letter-Word Identification, WJ III Applied Problems, WJ III Oral Comprehension, WJ III Spelling, WJ III Pre-Academic; "WJ III" is omitted hereafter for brevity)^32^ measured by child assessments, and three social-emotional outcomes (Behavior Problems, Social Skills, Social Competency) measured by parent interviews.

Cognitive outcomes were measured by one-on-one child assessments for 45 to 60 min.^6,7,33^ PPVT measures receptive vocabulary in standard English (Cronbach's α = 0.62–0.84). Oral Comprehension measures an ability to comprehend a short passage by listening and provide a missing word through reasoning (α = 0.76–0.89). Letter-Word Identification measures the ability to identify letters and words from a picture or isolated letters and words (α = 0.82–0.94). Spelling measures the ability to correctly spell spoken words (α = 0.70–0.94). Applied Problems measures an ability to analyze and solve math problems (α = 0.85–0.90). Pre-Academic is a composite measure of Letter-Word Identification, Applied Problems, and Spelling (α = 0.67–0.85). To reduce the time required to test the participating children, PPVT was adapted to create a shortened version using item response theory, and WJ III tests were subject to a rule that stopped the test when three consecutive items were incorrect. PPVT was scored with a marginal maximum likelihood estimation that is based on each child’s actual test scores and a prior distribution separately by the age cohorts estimated from all children in each cohort. The WJ III tests were measured in W-ability scores, a mathematical transformation of the Rasch model, which is based on item response theory. These scores for PPVT and WJ III were provided with the HSIS dataset.

Parent interviews were conducted for primary caregivers.^6,7,33^ Social Skills assesses social skills such as cooperative and emphatic behaviors and approaches to learning such as openness to new concepts, curiosity, and positive attitudes towards gaining knowledge (α = 0.57–0.85). Social Competency measures the ability to have social interactions (α = 0.50–0.94). Behavior Problems is a composite measure of aggressive, withdrawn, and hyperactive behaviors (α = 0.74–0.96). A more detailed description and a measurement method of each outcome are available in the HSIS official reports.^6,7,33^.

### Covariates

Although the HSIS was an RCT with no expected confounding, the HSIS official reports recommended covariate adjustment for two reasons^6,7,33^: 1) strong predictors of the outcome, such as sociodemographic variables and baseline outcomes, were included to enhance statistical precision; 2) baseline outcomes were included to account for any systematic bias at baseline. Following these recommendations, we adjusted for children’s sociodemographic variables and HSIS-related variables. Children’s sociodemographic variables included gender (male, female), race/ethnicity (White/other, Black, Hispanic), primary language at baseline (English, Spanish), special needs (yes, no), primary caregiver’s age (continuous), teen mom at birth (yes, no), living with a single parent (yes, no), recent immigrant parents (yes, no), parents’ marital status (not married, married, separated/divorced/widowed), parental education level (less than high school, high school graduates, beyond high school), urbanicity (urban, rural), household risk (low, moderate, high). Household risk index was developed by the researchers of the HSIS official reports based on five characteristics^6^: 1) receipt of TANF or Food Stamps, 2) both parents with education level less than high school, 3) both parents unemployed or not in education, 4) living with a single parent, 5) teen mom at birth. Three categories (low, moderate, high) were created by the number of these characteristics reported in the parent interview. HSIS-related variables included age cohort (age 3, age 4) and baseline outcomes (PPVT, Pre-Academic, Behavior Problems, Social Skills, and Social Competency).

### Statistical analysis

Sample characteristics were presented for the total sample and by treatment status. Primary analyses were performed on the 3-year-old cohort, the 4-year-old cohort, and the pooled cohort. Three-level multilevel models were fitted by specifying Head Start programs at level-3, centers at level-2, and children at level-1 to account for clustering at Head Start programs and centers. While multilevel models are generally fitted with the assumption that level-1 residuals are normally distributed with constant variance (i.e., homoscedasticity), we applied an extended version that models level-1 variance as a function of level-1 covariates. Such a variance modelling approach is called a complex (level-1) variance model.^27,34^ The primary analyses (Model 1) were specified as,

Model 1:

$$Y_{ijk} = \beta_{0} + \beta_{1} T_{ijk} + \beta X_{ijk}^{^{\prime}} + \left( {v_{0k} + u_{0jk} + e_{1ijk} T_{ijk} + e_{2ijk} C_{ijk} } \right)$$

Model 1 residual distribution:

$$\left[ {v_{{0k}} } \right]~\sim ~N\left( {0,\sigma _{{v_{0} }}^{2} } \right);\;[u_{0jk} ]~\sim ~N\left(0,\sigma _{{u_{0} }}^{2} \right);\;\left[ {\begin{array}{*{20}c} {e_{{1ijk}} } \\ {e_{{2ijk}} } \\ \end{array} } \right]~\sim ~N\left( {0,\left[ {\begin{array}{*{20}c} {\sigma _{{e_{1} }}^{2} } & {} \\ - & {\sigma _{{e_{2} }}^{2} } \\ \end{array} } \right]} \right)$$

where ${Y}_{ijk}$ is an outcome variable for child $i$ in center $j$ in program $k$, ${X}_{ijk}^{^{\prime}}$ is a vector of child-level covariates, ${T}_{ijk}$ is an indicator variable for the treatment group (i.e., Head Start), and ${C}_{ijk}$ is an indicator variable for the control group. All continuous covariates (baseline outcomes, primary caregiver’s age) were centered at their means for interpretability of regression coefficients. Total variance is partitioned into the program-level (${\sigma }_{{v}_{0}}^{2}$), the center-level (${\sigma }_{{u}_{0}}^{2}$), the child-level, and the child-level variance is further partitioned into treatment group variance (${\sigma }_{{e}_{1}}^{2}$) and control group variance (${\sigma }_{{e}_{2}}^{2}$). These two variance estimates are the main parameters of interest, and the equality of the variances was tested by F-test for normally distributed outcomes (PPVT, Letter-Word Identification, Applied Problems, Oral Comprehension, Spelling, Pre-Academics) and Levene’s test for the rest (Behavior Problems, Social Skills, Social Competency). A statistically significant difference between the two variances indicates that there may be a substantial amount of HTE, and more exploration should follow. The variance estimates were visualized in the 95% variation bounds, which indicate that 95% of the observations lie between the lower and upper bounds.^35^ They were calculated with the complex variance model estimates as follows: $mean \pm 1.96*\sqrt {child - level~variance~}$

Exploratory secondary analyses were conducted on the pooled cohort to investigate for which subgroups the treatment effects were meaningfully differential, and whether there remains HTE even after accounting for these treatment-subgroup interactions. Model 2 and 3 tested for the interactions between the treatment and a child’s primary language, parental education level, respectively, and for the difference in the treatment group variance and control group variance within each subgroup. Model 2 was specified as,

Model 2:

$$Y_{{ijk}} = \beta _{0} + \beta _{1} T_{{ijk}} + \beta _{2} S_{{ijk}} + ~\beta _{3} T_{{ijk}} S_{{ijk}} + \beta X_{{ijk}}^{'} + \left( {v_{{0k}} + u_{{0jk}} + e_{{1ijk}} S\left( T \right)_{{ijk}} + e_{{2ijk}} S\left( C \right)_{{ijk}} + e_{{3ijk}} E\left( T \right)_{{ijk}} + e_{{4ijk}} E\left( C \right)_{{ijk}} } \right)$$

Model 2 residual distribution:

$$[v_{0k}]~\sim ~N(0,\sigma _{{v_{0} }}^{2} );\;[u_{0jk} ]~\sim ~N(0,\sigma _{{u_{0} }}^{2} );\;\left[ {\begin{array}{*{20}c} {e_{{1ijk}} } \\ {e_{{2ijk}} } \\ {e_{{3ijk}} } \\ {e_{{4ijk}} } \\ \end{array} } \right]~\sim ~N\left( {0,\left[ {\begin{array}{*{20}c} {\sigma _{{e_{1} }}^{2} } & {} & {} & {} \\ - & {\sigma _{{e_{2} }}^{2} } & {} & {} \\ - & - & {\sigma _{{e_{3} }}^{2} } & {} \\ - & - & - & {\sigma _{{e_{4} }}^{2} } \\ \end{array} } \right]} \right)$$

where ${S}_{ijk}$ is an indicator variable for Spanish as a primary language, ${S(T)}_{ijk}$ and ${S(C)}_{ijk}$ are indicator variables for treatment and control groups among children with Spanish as a primary language, and ${E(T)}_{ijk}$ and ${E(C)}_{ijk}$ are indicator variables for treatment and control groups among children with English as a primary language. The parameter for interaction, ${\beta }_{3}$, between the treatment and the subgroup (i.e., Spanish as a primary language) is included to test for HTE across the subgroups, and the treatment group variance and control group variance are now separated into each subgroup (Spanish-Treatment: ${\sigma }_{{e}_{1}}^{2}$; Spanish-Control: ${\sigma }_{{e}_{2}}^{2}$; English-Treatment: ${\sigma }_{{e}_{3}}^{2}$; English-Control: ${\sigma }_{{e}_{4}}^{2}$). Within each subgroup, the treatment group variance and the control group variance are compared to check whether there is remaining HTE after accounting for the interactions between the treatment the subgroups. There are one more interaction parameter and two more variance parameters in Model 3 because the parental education level has three subgroups, one more than Model 2.

Loss to follow-ups occurred as with any longitudinal study. After applying list-wise deletions for children with missing data, we applied weights provided by the HSIS dataset to control for potential bias from differential loss to follow-ups by treatment status. The weights included the nonresponse probability to adjust for different response rates across demographic groups and the selection probability at every stage of sampling to ensure the model estimates reflect the parameters for a nationally representative Head Start sample. The weights were also used in the HSIS official reports. Descriptions of the weight construction are detailed in the HSIS official technical report.^33^ All models were fitted in R 4.0.0 using the R2MLwiN package to access MLwiN 3.04^36^ for multilevel modelling.

### Ethical approval

The HSIS data were not collected specifically for this study and no one on the study team has access to identifiers linked to the data. These activities do not meet the regulatory definition of human subject research. As such, an Institutional Review Board (IRB) review is not required. The Harvard Longwood Campus IRB allows researchers to self-determine when their research does not meet the requirements for IRB oversight via guidance online regarding when an IRB application is required using an IRB Decision Tool.

## Results

At baseline, the treatment group (n = 2,646) had a larger sample size than the control group (n = 1,796), which is consistent with the randomization design described above (Table 1). The percentage of missing data for each variable in the analyses ranged from 0 to 1.5%. A slightly higher proportion of children were Hispanics/other (36.0%) than White (33.7%) and Black (30.3%). About a quarter (25.7%) used Spanish as a primary language. Approximately half (50.4%) of children lived with a single biological parent, 84.3% lived in an urban setting, 16.9% had teen mothers at birth, and 12.8% had special needs. The average primary caregiver’s age was about 29, 38.0% of the children had mothers who did not graduate from high school, and 19.2% were recent immigrants. The treatment group was more likely to have special needs (13.8% vs. 11.4%; *p* = 0.018) and less likely to have had teen mothers at birth (15.9% vs. 18.4%; *p* = 0.038). Baseline means and variances were comparable between treatment and control groups for all outcomes except PPVT, which had a slightly lower mean score among the treatment group (*p* = 0.020). The response rates varied across the outcomes, ranging from 80.2 to 81.8% in 2003, 78.8 to 79.4% in 2004, 76.5 to 79.1% in 2005, and 71.3 to 75.3% in 2007–8 (Table A1).

**Table 1:** Sample characteristics at baseline by the treatment and control groups.

		Overall	Control	Head start	*p*-value	Missing
N		4442	1796	2646		
Age cohort (%)	3	2449 (55.1)	985 (54.8)	1464 (55.3)	0.773	0
	4	1993 (44.9)	811 (45.2)	1182 (44.7)		
Gender (%)	Male	2239 (50.4)	912 (50.8)	1327 (50.2)	0.704	0
Race/ethnicity (%)	White	1496 (33.7)	623 (34.7)	873 (33.0)	0.502	0
	Black	1348 (30.3)	536 (29.8)	812 (30.7)		
	Hispanic & others	1598 (36.0)	637 (35.5)	961 (36.3)		
Primary language (%)	English	3301 (74.3)	1345 (74.9)	1956 (73.9)	0.491	0
	Spanish	1141 (25.7)	451 (25.1)	690 (26.1)		
Parental education (%)	More	1274 (28.7)	505 (28.1)	769 (29.1)	0.558	0
	High school	1481 (33.3)	592 (33.0)	889 (33.6)		
	Less	1687 (38.0)	699 (38.9)	988 (37.3)		
Single parent (%)		2239 (50.4)	907 (50.5)	1332 (50.3)	0.940	0
Recent immigrant (%)		855 (19.2)	337 (18.8)	518 (19.6)	0.525	0
Marital status (%)	Married	1972 (44.4)	806 (44.9)	1166 (44.1)	0.882	0.1
	Separated & Divorced & Widowed	724 (16.3)	290 (16.1)	434 (16.4)		
	Never	1742 (39.2)	699 (38.9)	1043 (39.4)		
Special needs (%)		570 (12.8)	204 (11.4)	366 (13.8)	0.018	0
Teen mom (%)		752 (16.9)	330 (18.4)	422 (15.9)	0.038	0
Urban (%)		3746 (84.3)	1513 (84.2)	2233 (84.4)	0.927	0
Household risk (%)	Low	3383 (76.2)	1399 (77.9)	1984 (75.0)	0.081	0
	Moderate	741 (16.7)	277 (15.4)	464 (17.5)		
	High	318 (7.2)	120 (6.7)	198 (7.5)		
Caregiver’s age (mean (SD))		28.91 (7.34)	28.65 (7.06)	29.08 (7.52)	0.057	0
PPVT (mean (SD))		248.21 (42.64)	250.03 (42.76)	246.97 (42.53)	0.020	1.5
Pre-Academic (mean (SD))		347.27 (22.99)	346.75 (22.82)	347.61 (23.11)	0.225	1.5
Behavior Problems (mean (SD))		6.15 (3.65)	6.21 (3.68)	6.11 (3.62)	0.330	0
Social skills (mean (SD))		12.25 (1.79)	12.25 (1.77)	12.25 (1.80)	0.590	0
Social competency (mean (SD))		10.79 (1.45)	10.80 (1.44)	10.78 (1.46)	0.437	0

**Figure 2:**
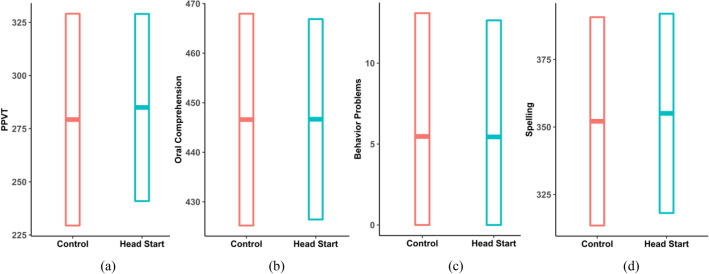
Visualized examples of the outcome distribution comparison between the Head Start and Control groups. The plot (**a**), (**b**), (**c**), and (**d**) visualize the outcome distributions for PPVT in the first year after Head Start, Oral Comprehension in the first year, Behavior Problems in the third grade year, and Spelling in the first year, respectively. The centered line is the mean of the outcome, and the surrounding bars are the 95% variation bounds, describing how variable the data are.

Three combinations of the effect on the mean and variance (i.e., mean and variance for the Head Start children vs. the Control children) are observed from the complex variance model results: 1) increase in the mean, decrease in the variance (Fig. 2a); 2) increase in the mean, no change in the variance (Fig. 2c, d); 3) no change in the mean, decrease in the variance (Fig. 2b). An increase in the mean reflects improvement for the outcomes except in the case of Behavior Problems for which a decrease would mean improvement. In both scenario 1) and 2) for the main analysis (i.e., Model 1), Head Start increased the mean, indicating that Head Start improves the outcomes on average. In scenario 1), a decrease in the variance that was accompanied with an increase in the mean suggests that the improvement may have been larger for those at the lower tail of the outcome distribution. In scenario 3), Head Start did not change the mean on average, but a decrease in the variance suggests that some were benefitted or harmed. Further exploration of HTE is needed. In the subgroup analyses (i.e., Model 2, 3, and 4), if the variance change observed in Model 1 disappeared with statistically significant interactions, the treatment-subgroup interactions may have explained away the HTE observed in Model 1. If the variance change persisted, on the other hand, further stratification within the subgroups with the variance change may be able to explain the HTE.

### Outcomes with increased mean and decreased variance

The pooled cohort analyses showed that PPVT, Letter-Word Identification, Applied Problems, and Pre-Academic had the pattern of increased mean and decreased variance for the Head Start children compared to the Control children (Table 2). For the four cognitive outcomes, Head Start had short-term effects that did not last beyond the third year. For example, the Head Start children scored higher on PPVT until the third year after Head Start but the effect was attenuated with time (1st year: β [SE] = 5.69 [0.90], *p* < 0.001; 2nd year: β [SE] = 2.09 [1.08], *p* = 0.051; 3rd year: β [SE] = 2.00 [0.77], *p* = 0.009). The effects on the mean were often accompanied with the effects on the variance. For example, the Head Start children had smaller variance of PPVT until the second year after Head Start (1st year: δ = − 21.90, *p* < 0.001; 2nd year: δ = − 13.65, *p* = 0.051). The visualization suggests that those at the lower part of the outcome distribution may have benefitted more (Fig. 2a). When the cohorts were analyzed separately, the pattern of increased mean and decreased variance persisted for the four cognitive outcomes at most follow-ups (Tables A2 and A3). At a few time points, the change in variance was statistically insignificant, but had the consistent direction and magnitude, indicating loss of power. At second and third year of follow-ups, the increased mean was only observed for the 3-year-old cohort.

**Table 2:** The effect of Head Start on the means and variances for cognitive and social-emotional outcomes for the pooled cohort.

		1st year		2nd year		3rd year		3rd grade	
		Estimate	*p*-value	Estimate	*p*-value	Estimate	*p*-value	Estimate	*p*-value
PPVT	Difference in mean^a^	**5.69** **(0.90)**	< 0.001	2.09 (1.08)	0.051	**2.00** **(0.77)**	0.009	1.44 (0.87)	0.096
	% change in variance^2^^b^	− **21.90**	< 0.001	− **13.65**	0.007	1.18	0.836	− 4.23	0.456
Letter-Word Identification	Difference in mean	**5.47** **(1.02)**	< 0.001	1.72 (0.99)	0.081	0.29 (1.15)	0.801	1.24 (0.99)	0.210
	% change in variance	− 0.23	0.955	− **17.45**	0.001	1.32	0.851	− 0.42	0.960
Applied Problems	Difference in mean	**3.47** **(1.01)**	< 0.001	**1.89** **(0.66)**	0.004	0.32 (0.70)	0.644	− 0.36 (0.68)	0.596
	% change in variance	− **16.79**	0.029	− **19.48**	0.004	− 6.81	0.353	− 3.12	0.737
Oral Comprehension	Difference in mean	0.06 (0.37)	0.862	0.44 (0.44)	0.317	0.80 (0.52)	0.122		
	% change in variance	− **10.47**	0.045	1.74	0.760	− 5.83	0.328		
Spelling	Difference in mean	**2.96** **(0.69)**	< 0.001	0.97 (0.92)	0.290	0.55 (0.85)	0.518		
	% change in variance	− 8.51	0.202	-3.35	0.620	− 0.50	0.950		
Pre-Academic	Difference in mean	**3.91** **(0.71)**	< 0.001	**1.45** **(0.72)**	0.043	0.41 (0.76)	0.588		
	% change in variance	− 11.34	0.109	− **18.35**	0.002	− 0.92	0.897		
Behavior Problems	Difference in mean	− 0.20 (0.11)	0.082	− 0.11 (0.11)	0.312	0.01 (0.11)	0.955	− 0.03 (0.14)	0.861
	% change in variance	1.12	0.863	− 3.61	0.633	− 9.74	0.090	− **10.70**	0.040
Social Skills	Difference in mean	− 0.02 (0.06)	0.733	0.01 (0.04)	0.755	0.10 (0.06)	0.075	0.04 (0.06)	0.457
	% change in variance	− 0.73	0.922	− 11.02	0.139	− 3.77	0.571	1.88	0.753
Social Competency	Difference in mean	− 0.01 (0.05)	0.812	− 0.01 (0.04)	0.792	0.03 (0.04)	0.417		
	% change in variance	5.49	0.540	3.00	0.742	− 8.18	0.384		

Point estimates with *p*-value less than 0.05 are bolded.

^a^ difference in mean is calculated by $mean\;\left( {Head\;Start} \right) - mean\;(Control)$.

^b^% change in variance is calculated by$\frac{{var\;\left( {Head\;Start} \right) - var\;\left( {Control} \right)}}{{var\;\left( {Control} \right)}}*\;100$.

For PPVT, Applied Problems, and Pre-Academic, subgroup analyses revealed that larger effects for children with Spanish as a primary language or with low parental education level can partly explain the Head Start effect on the variance in Model 1. For example, Head Start had a consistently larger effect on PPVT for children with Spanish as a primary language, which was statistically significant even in the third grade year (β [SE] = 4.89 [1.85], *p* = 0.008). After taking the interactions into account, the variance for the Spanish-Head Start group was smaller in the first and second years after Head Start (1st year: δ = − 21.70, *p* = 0.032; 2nd year: δ = − 34.00, *p* < 0.001) compared to the Spanish-Control group, whereas the variance for the English-Head Start group was 21.06% smaller only in the first year (*p* < 0.001) (Table A1). No statistically significant interactions were observed across parental education levels, but Head Start reduced the variance of the Head Start group with parents with high school as the highest education level in the first year (δ = − 27.96, *p* < 0.001) and those with less than high school in the first and second years (1^st^ year: δ = − 23.23, *p* = 0.003; 2nd year: δ = − 20.37, *p* = 0.008) (Table A2).

### Outcomes with no change in the mean and decreased variance

For Oral Comprehension and Behavior Problems, Head Start did not change the mean but reduced the variance of children’s scores (Table 2). In the first year after Head Start, the Head Start children had the variance of Oral Comprehension that was 10.47% lower than the Control children (*p* = 0.045). Both tails of the outcome distribution shrunk toward the mean (Fig. 2c). No interactions explained the reduced variance in the first year, but the reduced variance was observed only for the children that had parents with less than high school education (δ = − 17.97, *p* < 0.044) (Table A4). In the second year, Head Start had a negative effect for children that had parents with high school as the highest education (β [SE] = − 2.25 [0.97], *p* = 0.020). For Behavior Problems, in the third grade year, the Head Start children had the variance 10.70% lower than the Control children (*p* = 0.04) (Table 2). Because the scores of Behavior Problems cannot be lower than zero, the reduced variance was due to the higher tail of the outcome distribution shifted down (Fig. 2d). The reduced variance was not explained by the tested interactions and found even within children who use Spanish as a primary language (δ = − 15.17, *p* < 0.043) (Table A5) or had parents with high school as the highest education (δ = − 19.80, *p* < 0.010) (Table A4).

For Oral Comprehension and Behavioral Problems, the pattern for the mean and variance was consistent at most follow-ups when the cohorts were analyzed separately (Tables A2 and A3). For Oral Comprehension at the first follow-up, the variance change for the 3-year-old cohort was not statistically significant, but its direction and magnitude was consistent, indicating loss of power. For Behavioral Problems at the first and second follow-ups, the 3-year-old cohort experienced decreased mean (i.e., reduced behavioral problems; positive effect), which was masked in the pooled cohort analyses.

### Outcomes with no change in the variance

For Spelling, there was a pattern of an increased mean for the Head Start children without a change in the variance. In the first year after Head Start, the Head Start children scored higher on average (β [SE] = 2.96 [0.69], *p* < 0.001), but the effect faded away in the later years (Table 2). The entire outcome distribution shifted upwards without a substantial change in the variance (Fig. 2b). For Social Skills and Social Competency, there was no consistent pattern of change in either the mean or the variance across all follow-up years (Table 2). For Spelling, Social Skills, and Social Competency, the pattern for the mean and variance was consistent when the cohorts were analyzed separately (Tables A2 and A3).

## Discussion

We applied complex variance modelling using the HSIS data to examine HTE of Head Start, in addition to ATE. Head Start had positive short-term effects on the means of multiple cognitive outcomes, while having no effect on the means of social-emotional outcomes. Modelling variance by treatment status revealed that Head Start reduced the variances of multiple cognitive and one social-emotional outcomes, meaning that substantial HTE exits. In particular, the increased mean and the decreased variance reflect the ability of Head Start to improve the outcomes while reducing their variability. The reduced variances were partly explained by the larger benefits for children with Spanish as a primary language or low parental education level, suggesting that at least some parts of the reduced variances reflect the reduced social inequalities in the outcomes. Interestingly, even after accounting for these treatment-subgroup interactions, the HTE remained for some outcomes, and their variances were reduced even within these subgroups. For multiple outcomes at certain follow-up years, the effects on the variance were present even when the effects on the mean were null. Without modelling variance, such an HTE is likely to have been masked by the non-significant effect on average.

Consistent with the HSIS official reports, Head Start improved several cognitive outcomes at the first and second years, but the effects faded away at later follow-ups.^6,7^ We additionally showed that the variances of these outcomes were also reduced for the Head Start children compared to the Control children. With the comparable variances at baseline, the difference in the post-treatment variances suggests that there was a meaningful amount of HTE that should be further investigated. In particular, the reduction in the variance with the increased mean may mean that Head Start was able to pull those at the lower part of the outcome distribution upwards to the mean. Indeed, previous studies found that Head Start was more effective at improving cognitive outcomes for many high-risk subgroups, including children with Spanish as a primary language,^12,13^ lower cognitive test scores at baseline,^12^ non-parental care at baseline,^15^ low and moderate parental pre-academic stimulation,^37^ or special needs.^19^ Similarly, we found that larger benefits for children with Spanish as a primary language or a low parental education level appeared to explain away some of the effects on the variance. Head Start may have been more effective on cognitive outcomes for these children because it offered academic resources, which their home environments may have lacked, for developing English language skills and cognitive abilities. However, even after accounting for these treatment-subgroup interactions, the Head Start children within these subgroups had smaller variability than the Control children. After Head Start, in other words, the outcome distributions of even these high-risk subgroups shrunk, indicating that substantial HTE exists within these subgroups. Particularly, those scored lower within these subgroups appeared to have benefitted more, further suggesting the compensatory effects of Head Start. If statistical power allows, finer stratification may be able to uncover for whom Head Start was effective among children with Spanish as a primary language or a low parental education level.

No clear pattern of the effects on the mean were observed for the social-emotional outcomes, except that the 3-year-old cohort experienced short-term positive effects on Behavioral Problems. Even the subgroup analyses did not find a clear pattern for the effects on the mean. Previous studies have also investigated heterogeneous effects on social-emotional outcomes for children who had foster care at baseline^38^ and who had experienced violence,^39^ but found no effects on the mean. Despite the absence of meaningful ATE, the Head Start children had smaller variances for one social-emotional outcome, Behavior Problems, and one cognitive outcome, Oral Comprehension, suggesting there are subgroups with heterogeneous effects for these outcomes. In this case, since the ATE was null, comparing the outcome distributions of the Head Start and Control groups by visualization helped understand the effects. For Oral Comprehension, the distribution shrunk from both tails, suggesting that there may have been subgroups that experienced negative impacts, as well as subgroups with positive impacts. For Behavior Problems, the distribution shrunk from the higher tail, meaning that there were positive effects for certain subgroups because a lower score means a better outcome for Behavior Problems. The positive effect in the 3-year-old cohort may explain such a distributional shift. The smaller variances were observed within children with Spanish as a primary language or children of parents with high school as the highest education level. Further exploration among these subgroups may reveal for which subgroup Head Start worked well.

Findings that Head Start improved multiple outcomes on average and reduced their variance are especially important because the program had an additional goal of shrinking the outcome distribution. The reduced variance on cognitive outcomes may be transferred further to academic performances. Indeed, previous observational studies found that Head Start decreased grade repetition rates, while increasing high school graduation rate and college attendance, which are signs of reduced outcome distribution by improving at the lower tail.^40,41^ If the HSIS participants were tracked in their adulthood, the Head Start effect on the mean and variance of their adulthood outcomes such as income also could be evaluated.

One strength of our study is the use of multilevel models to adjust for clustering among Head Start programs and centers. Partitioning variance at program-, center-, and child-level gives more valid estimates of variance and is especially important when variance estimates are the parameters of primary interest. Another strength is the use of the RCT data. While analytical approaches to modelling individual variability have been extended to quasi-experimental^28^ and cross-sectional observational studies,^42^ a well-designed RCT remains the most appropriate setting to estimate the treatment effect on variance because treatment and control groups are expected to be exchangeable at baseline. In HSIS, the treatment group had a larger sample size than the control group, but this difference does not alone explain the observed variance differences; no identical pattern was found across all outcomes. When the sample size is large enough to represent the population variance, the difference in sample size between the two groups would not drive the difference in variance estimates.

Our study has limitations. First, our analysis excluded categorical outcomes because only continuous outcomes fit with our framework of comparing variances and visualizing them as distributions. Especially for binary outcomes, extending this complex level-1 variance modelling approach is not very straightforward because level-1 variance in a multilevel logistic regression model is assumed to come from a logistic distribution with a fixed variance of π^2^/3.^25^ Nonetheless, future studies should utilize methods that can reveal HTE for categorical outcomes beyond what is possible with a single covariate interaction analysis, such as latent class analysis^43^ and intersectional multilevel analysis.^44,45^ Second, the treatment effect on variance is a summary statistic of the overall outcome distribution and does not identify for whom exactly Head Start worked. For example, when Head Start increased a cognitive outcome on average and reduced variance by shifting up those at the lower tail of the outcome distribution, we interpreted that Head Start improved those at the lower tail more than others. This is only true under the rank preservation assumption, in which children keep their ranks in the outcome distribution regardless of the treatment status. Although the assumption is untestable, we found that some subgroups that scored lower before were benefitted more, which provide support for our interpretation.

Given that children experience multiple social identities and environments simultaneously, it is no surprise to see HTE even within subgroups like children with low parental education level.^46,47^ However, analysis of HTE often terminates at a single covariate stratification, offering a limited aspect of HTE. Individual variability around the averages is often disregarded. In an RCT setting, we demonstrated that modelling post-treatment variances can enrich interpretations of a treatment effect in two major ways. First, a substantial difference in variances between treatment and control groups can motivate further investigation to better understand for whom the treatment works. Second, the magnitude and direction of the effect on variance can suggest which part of the outcome distribution had heterogeneous effects. Routinely monitoring the treatment effects on variances of the outcomes, in addition to the means, would lead to a more comprehensive program evaluation that describes how a program performs on average and on the entire distribution.

## Supplementary Information

Supplementary Information.

## Data Availability

The Head Start Impact Study data are hosted by Inter-university Consortium for Political and Social Research. Restrictions apply to the availability of these datasets. All methods were carried out in accordance with relevant guidelines and regulations.
