# Supplementary material for: Assessment of heterogeneous Head Start treatment effects on cognitive and social-emotional outcomes
Source: Sci Rep. 2022 Apr 19;12:6411. doi: 10.1038/s41598-022-10192-1 (PMC9018838; doi:10.1038/s41598-022-10192-1)
Supplement: Supplementary file 1 — Supplementary Information. [file 41598_2022_10192_MOESM1_ESM.pdf]

Title
Assessment of heterogeneous Head Start treatment effects on cognitive and social-emotional outcomes

Authors and Affiliations
Sun Yeop Lee[a], Rockli Kim[b,c,d,*], Justin Rodgers[d], S.V. Subramanian[d,e]

[a] Department of Epidemiology, Harvard T.H. Chan School of Public Health, Boston, MA, USA.
[b] Division of Health Policy and Management, College of Health Sciences, Korea University, Seoul, South Korea
[c] Interdisciplinary Program in Precision Public Health, Department of Public Health Sciences, Graduate School of Korea University, Seoul, South Korea
[d] Harvard Center for Population & Development Studies, Cambridge, MA, USA.
[e] Department of Social and Behavioral Sciences, Harvard T.H. Chan School of Public Health, Boston, MA, USA.

Supplementary File

Table A. 1 Sample size by follow-up year and outcome

Table A. 2 The effect of Head Start on the means and variances of cognitive and social-emotional outcomes for the 3-year-old cohort

Table A. 3 The effect of Head Start on the means and variances of cognitive and social-emotional outcomes for the 4-year-old cohort

Table A. 4 The effect of Head Start on the means and variances of cognitive and social-emotional outcomes by parental educational level

Table A. 5 The effect of Head Start on the means and variances of cognitive and social-emotional outcomes by a child's primary language

Table A. 1 Sample size by follow-up year and outcome

	2003	2004	2005	2007-8
PPVT	3621 (81.5)	3518 (79.2)	3398 (76.5)	3168 (71.3)
Letter-Word Identification	3627 (81.6)	3517 (79.2)	3396 (76.5)	3169 (71.3)
Applied Problems	3601 (81.1)	3500 (78.8)	3397 (76.5)	3169 (71.3)
Oral Comprehension	3563 (80.2)	3507 (79.0)	3396 (76.5)	n/a
Spelling	3635 (81.8)	3520 (79.2)	3395 (76.4)	n/a
Pre-Academic	3594 (80.9)	3499 (78.8)	3393 (76.4)	n/a
Behavior Problems	3632 (81.8)	3526 (79.4)	3512 (79.1)	3342 (75.2)
Social Skills	3632 (81.8)	3527 (79.4)	3512 (79.1)	3342 (75.2)
Social Competency	3633 (81.8)	3527 (79.4)	3513 (79.1)	n/a

Note. The sample size at baseline (i.e., Year 2002) was 4371 for cognitive measures and 4438 for social-emotional measures after list-wise deletions for participants with missing data at baseline (71 for cognitive measures, 4 for social-emotional measures).

Table A. 2 The effect of Head Start on the means and on the distribution for cognitive and social-emotional outcomes for the 3-year-old cohort

		1st year		*2nd year*		*3rd year*		*3rd grade*	
		estimate	p-value	estimate	p-value	estimate	p-value	estimate	p-value
PPVT	Difference in mean[1]	**6.50 (1.27)**	< 0.001	**2.87 (1.37)**	0.036	0.89 (0.95)	0.353	1.88 (0.99)	0.057
	% change in variance[2]	**-22.13**	< 0.001	**-11.63**	0.051	-1.67	0.840	-11.60	0.181
Letter-Word Identification	Difference in mean	**5.81 (1.20)**	< 0.001	**3.25 (1.09)**	0.003	-0.15 (1.33)	0.908	0.94 (1.14)	0.411
	% change in variance	0.46	0.929	-8.30	0.187	9.42	0.323	4.22	0.755
Applied Problems	Difference in mean	**4.14 (1.01)**	0.002	**2.81 (0.82)**	< 0.001	-0.11 (0.84)	0.894	-0.19 (0.88)	0.826
	% change in variance	-13.33	0.090	-16.85	0.07	1.43	0.881	-6.09	0.646
Oral Comprehension	Difference in mean	0.51 (0.43)	0.233	1.02 (0.55)	0.064	1.15 (0.64)	0.072		
	% change in variance	-10.32	0.186	-0.09	0.992	-7.55	0.322		
Spelling	Difference in mean	**3.20 (0.93)**	< 0.001	2.09 (1.12)	0.063	0.29 (0.94)	0.759		
	% change in variance	-12.72	0.153	7.17	0.411	8.14	0.479		
Pre-Academic	Difference in mean	**4.31 (0.90)**	< 0.001	**2.63 (0.78)**	< 0.001	0.03 (0.83)	0.974		
	% change in variance	-10.65	0.161	-6.68	0.457	13.06	0.211		
Behavior Problems	Difference in mean	**-0.38 (0.14)**	0.008	**-0.42 (0.17)**	0.011	-0.08 (0.61)	0.611	0.05 (0.20)	0.786
	% change in variance	-6.74	0.435	-16.31	0.077	-15.16	0.054	-10.55	0.152
Social Skills	Difference in mean	0.01 (0.07)	0.921	0.06 (0.06)	0.377	0.14 (0.08)	0.057	**0.173 (0.07)**	0.021
	% change in variance	-1.81	0.839	-20.80	0.070	-11.35	0.217	-2.56	0.748
Social Competency	Difference in mean	-0.02 (0.06)	0.707	-0.01 (0.06)	0.820	0.07 (0.05)	0.187		
	% change in variance	4.45	0.705	-3.21	0.807	-17.46	0.153		

Note. Point estimates with p-value less than 0.05 are bolded.

[1] difference in mean is calculated by $mean(Head\ Start) - mean(Control)$

[2] % change in variance is calculated by $\frac{var(Head\ Start) - var(Control)}{var(Control)} * 100$

Table A. 3 The effect of Head Start on the means and on the distribution for cognitive and social-emotional outcomes for the 4-year-old cohort

		1st year		*2nd year*		*3rd year*		*3rd grade*	
		estimate	p-value	estimate	p-value	estimate	p-value	estimate	p-value
PPVT	Difference in mean[1]	**4.39 (1.24)**	< 0.001	0.35 (1.41)	0.804	**3.02 (1.27)**	0.018	0.81 (1.45)	0.577
	% change in variance[2]	**-24.93**	< 0.001	**-15.58**	0.038	3.67	0.661	2.55	0.759
Letter-Word Identification	Difference in mean	**4.89 (1.29)**	< 0.001	-1.30 (1.66)	0.434	-0.13 (1.83)	0.944	0.96 (1.59)	0.547
	% change in variance	3.44	0.663	-15.41	0.063	-2.82	0.756	-4.90	0.590
Applied Problems	Difference in mean	**2.73 (1.15)**	0.018	0.22 (1.07)	0.837	0.84 (0.02)	0.347	-0.66 (0.94)	0.484
	% change in variance	**-23.38**	0.022	**-20.64**	0.061	-14.57	0.170	-0.91	0.905
Oral Comprehension	Difference in mean	-0.60 (0.34)	0.341	-0.38 (0.73)	0.600	-0.05 (0.75)	0.951		
	% change in variance	**-14.72**	0.029	2.01	0.770	-2.24	0.828		
Spelling	Difference in mean	**2.45 (0.99)**	0.013	-1.19 (0.36)	0.359	0.23 (1.26)	0.853		
	% change in variance	-5.24	0.587	-4.21	0.689	-5.20	0.583		
Pre-Academic	Difference in mean	**3.28 (0.85)**	< 0.001	-0.82 (1.15)	0.476	0.34 (1.17)	0.773		
	% change in variance	-11.56	0.25	**-19.60**	0.021	-10.35	0.258		
Behavior Problems	Difference in mean	0.20 (0.17)	0.907	**0.31 (0.14)**	0.026	0.16 (0.15)	0.286	-0.12 (0.18)	0.507
	% change in variance	10.44	0.261	20.39	0.055	-3.07	0.718	-9.49	0.179
Social Skills	Difference in mean	-0.06 (0.08)	0.468	-0.04 (0.07)	0.574	0.01 (0.08)	0.939	-0.11 (0.09)	0.249
	% change in variance	1.54	0.878	9.06	0.409	9.53	0.233	10.18	0.246
Social Competency	Difference in mean	0.01 (0.05)	0.984	-0.01 (0.05)	0.943	-0.03 (0.06)	0.640		
	% change in variance	8.81	0.403	17.13	0.165	10.59	0.414		

Note. Point estimates with p-value less than 0.05 are bolded.
[1] difference in mean is calculated by $mean(Head\ Start) - mean(Control)$
[2] % change in variance is calculated by $\frac{var(Head\ Start) - var(Control)}{var(Control)} * 100$

Table A. 4 The effect of Head Start on the means and variances of cognitive and social-emotional outcomes by parental educational level

		2003		*2004*		*2005*		*2007*	
		estimate	p-value	estimate	p-value	estimate	p-value	estimate	p-value
PPVT	HS	**6.12**	< .001	0.76	.659	1.80	.133	2.10	.137
	(ref: more than high school)	**(1.40)**		(1.73)		(1.20)		(1.42)	
	HS * (high school)	-2.05	.298	-1.02	.699	-1.85	.276	-2.50	.249
		(1.97)		(2.66)		(1.69)		(2.17)	
	HS * (less than high school)	0.59	.769	4.32	.079	2.07	.247	0.39	.823
		(2.04)		(2.46)		(1.79)		(1.76)	
	% change in variance	-11.29	.190	-6.86	.530	17.42	.130	-7.91	.465
	(more than high school)								
	% change in variance	**-27.96**	< .001	-8.79	.308	3.22	.751	2.84	.783
	(high school)								
	% change in variance	**-23.23**	.003	**-20.37**	.008	-7.56	.377	-6.47	.557
	(less than high school)								
Letter-Word	HS	**5.61**	< .001	2.56	.160	0.74	.687	1.17	.480
Identification	(ref: more than high school)	**(1.52)**		(1.82)		(1.85)		(1.65)	
	HS * (high school)	-1.81	.363	-2.36	.263	-2.28	.344	-2.34	.260
		(1.99)		(2.11)		(2.42)		(2.08)	
	HS * (less than high school)	1.16	.539	-0.13	.957	0.73	.768	2.04	.304
		(1.90)		(2.49)		(2.50)		(1.99)	
	% change in variance	8.64	.311	-11.14	.267	-4.08	.768	-6.71	.535
	(more than high school)								
	% change in variance	-3.09	.683	-12.15	.154	2.02	.859	7.75	.683
	(high school)								
	% change in variance	-2.88	.748	**-23.62**	.001	4.83	.610	-3.18	.758
	(less than high school)								
Applied	HS	1.35	.255	1.79	.110	-1.30	.262	-0.89	.427
Problems	(ref: more than high school)	(1.19)		(1.12)		(1.16)		(1.12)	
	HS * (high school)	0.41	.792	-0.65	.660	1.53	.285	0.59	.676
		(1.58)		(1.49)		(1.43)		(1.42)	
	HS * (less than high school)	**5.09**	.016	0.80	.648	2.87	.079	0.86	.573
		**(2.12)**		(1.76)		(1.63)		(1.53)	
	% change in variance	-8.86	.482	-2.94	.836	7.80	.419	-15.92	.438
	(more than high school)								
	% change in variance	4.17	.683	0.79	.948	2.34	.877	4.72	.820
	(high school)								
	% change in variance	**-29.14**	.003	**-34.74**	< .001	**-19.11**	.045	-0.63	.960
	(less than high school)								
Oral	HS	0.60	.318	1.09	.168	0.69	.397		
Comprehension	(ref: more than high school)	(0.60)		(0.79)		(0.82)			
	HS * (high school)	-1.32	.084	**-2.25**	.020	-0.80	.422		
		(0.76)		**(0.97)**		(1.00)			
	HS * (less than high school)	-0.28	.767	0.25	.793	0.93	.317		
		(0.94)		(0.98)		(0.94)			
	% change in variance	-7.34	.490	6.03	.546	-12.85	.266		
	(more than high school)								
	% change in variance	-6.72	.515	0.87	.914	4.98	.614		
	(high school)								
	% change in variance	**-17.97**	.044	0.90	.910	-8.53	.325		
	(less than high school)								
Spelling	HS	1.54	.126	0.69	.692	1.16	.392		
	(ref: more than high school)	(1.00)		(1.76)		(1.35)			
	HS * (high school)	1.00	.555	0.01	.994	-3.08	.111		
		(1.70)		(2.06)		(1.93)			
	HS * (less than high school)	**2.79**	.028	0.69	.756	1.04	.575		
		**(1.27)**		(2.25)		(1.87)			
	% change in variance	-6.86	.450	4.96	.715	0.04	.997		
	(more than high school)								
	% change in variance	-14.69	.213	-3.95	.740	24.34	.170		
	(high school)								
	% change in variance	-3.97	.704	-8.59	.397	-15.40	.197		
	(less than high school)								
Pre-Academic	HS	**2.91**	< .001	1.65	.193	0.21	.853		
	(ref: more than high school)	**(0.87)**		(1.26)		(1.16)			
	HS * (high school)	-0.36	.783	-1.06	.468	-1.33	.395		
		(1.31)		(1.47)		(1.57)			
	HS * (less than high school)	**2.88**	.019	0.41	.818	1.62	.313		
		**(1.23)**		(1.79)		(1.61)			
	% change in variance	-2.78	.808	-2.79	.814	4.15	.739		
	(more than high school)								
	% change in variance	-10.92	.235	-14.15	.140	7.00	.585		
	(high school)								
	% change in variance	-14.50	.173	**-27.68**	< .001	-7.66	.404		
	(less than high school)								
Behavior	HS	-0.15	.394	0.08	.696	-0.07	.747	-0.01	.950
Problems	(ref: more than high school)	(0.18)		(0.20)		(0.23)		(0.28)	
	HS * (high school)	-0.06	.759	-0.23	.395	0.26	.456	0.05	.896
		(0.21)		(0.27)		(0.35)		(0.39)	
	HS * (less than high school)	-0.05	.850	-0.29	.298	-0.01	.962	-0.05	.861
		(0.25)		(0.28)		(0.30)		(0.31)	

	% change in variance<br>(more than high school)	0.19	.988	3.35	.783	-21.44	.140	-15.04	.162
	% change in variance<br>(high school)	-7.70	.475	-6.89	.608	-6.20	.559	**-19.80**	.010
	% change in variance<br>(less than high school)	9.70	.271	-4.36	.620	-4.10	.601	0.40	.966
Social Skills	HS<br>(ref: more than high school)	0.01<br>(0.08)	.854	0.08<br>(0.08)	.292	0.14<br>(0.09)	.127	**0.25**<br>**(0.10)**	.017
	HS * (high school)	0.01<br>(0.13)	.927	-0.18<br>(0.12)	.132	-0.10<br>(0.14)	.473	-0.23<br>(0.16)	.155
	HS * (less than high school)	-0.09<br>(0.12)	.430	-0.03<br>(0.10)	.753	-0.03<br>(0.12)	.811	**-0.33**<br>**(0.13)**	.011
	% change in variance<br>(more than high school)	-1.57	.904	-3.45	.807	-11.91	.276	-0.77	.949
	% change in variance<br>(high school)	-4.11	.700	-11.04	.426	10.05	.478	3.45	.756
	% change in variance<br>(less than high school)	5.21	.712	-13.27	.306	-8.24	.346	2.73	.758
Social<br>Competency	HS<br>(ref: more than high school)	0.04<br>(0.07)	.536	-0.07<br>(0.06)	.247	-0.04<br>(0.06)	.494		
	HS * (high school)	-0.06<br>(0.10)	.559	0.08<br>(0.10)	.429	0.19<br>(0.10)	.060		
	HS * (less than high school)	-0.09<br>(0.10)	.397	0.08<br>(0.08)	.331	0.03<br>(0.10)	.754		
	% change in variance<br>(more than high school)	-28.40	.115	22.65	.194	16.05	.242		
	% change in variance<br>(high school)	18.24	.198	-6.79	.622	-19.44	.258		
	% change in variance<br>(less than high school)	23.51	.135	5.07	.708	-8.99	.597		

Table A. 5 The effect of Head Start on the means and variances of cognitive and social-emotional outcomes by a child's primary language

		2003		*2004*		*2005*		*2007*	
		estimate	p-value	estimate	p-value	estimate	p-value	estimate	p-value
PPVT	HS (ref: English)	**3.38 (0.94)**	< 0.001	0.04 (1.02)	0.965	1.19 (0.82)	0.145	0.15 (0.90)	0.863
	HS * Spanish	**9.17 (1.92)**	< 0.001	**8.12 (2.40)**	< 0.001	3.07 (1.71)	0.073	**4.89 (1.85)**	0.008
	% change in variance (Spanish)	**-21.70**	0.032	**-34.00**	< 0.001	-0.98	0.924	-7.71	0.438
	% change in variance (English)	**-21.06**	< 0.001	-3.61	0.530	2.28	0.751	-2.33	0.735
Letter-Word Identification	HS (ref: English)	**5.36 (0.99)**	< 0.001	1.28 (1.00)	0.199	-0.48 (1.23)	0.695	0.77 (1.16)	0.503
	HS * Spanish	0.36 (2.15)	0.864	1.72 (2.31)	0.455	2.89 (3.57)	0.418	1.79 (2.63)	0.494
	% change in variance (Spanish)	0.54	0.960	**-26.55**	0.010	1.85	0.882	-24.60	0.175
	% change in variance (English)	-0.94	0.847	**-13.47**	0.009	1.06	0.898	12.67	0.162
Applied Problems	HS (ref: English)	1.30 (0.73)	0.074	0.61 (0.62)	0.326	-1.03 (0.75)	0.171	-0.96 (0.80)	0.226
	HS * Spanish	**8.61 (2.66)**	0.001	**5.11 (1.69)**	0.002	**5.13 (1.52)**	< 0.001	2.31 (1.86)	0.214
	% change in variance (Spanish)	**-25.70**	0.025	**-30.00**	0.001	**-34.12**	0.008	-9.24	0.699
	% change in variance (English)	-10.28	0.162	-13.54	0.117	10.33	0.235	-0.07	0.992
Oral Comprehension	HS (ref: English)	-0.12 (0.44)	0.775	0.17 (0.43)	0.678	0.38 (0.55)	0.496		
	HS * Spanish	0.78 (0.78)	0.317	1.12 (1.30)	0.386	1.59 (1.27)	0.210		
	% change in variance (Spanish)	6.29	0.690	-0.18	0.986	-13.99	0.154		
	% change in variance (English)	**-13.29**	0.020	1.28	0.850	-1.32	0.824		
Spelling	HS (ref: English)	**2.07 (0.78)**	0.008	0.33 (1.08)	0.759	-0.43 (0.96)	0.655		
	HS * Spanish	3.44 (1.76)	0.051	2.55 (1.74)	0.142	3.68 (1.93)	0.056		
	% change in variance (Spanish)	-23.57	0.103	4.42	0.826	-20.73	0.148		
	% change in variance (English)	-2.72	0.701	-5.04	0.390	9.61	0.272		
Pre-Academic	HS (ref: English)	**2.89 (0.57)**	< 0.001	0.65 (0.75)	0.384	-0.63 (0.83)	0.444		
	HS * Spanish	**4.01 (1.81)**	0.026	**3.18 (1.58)**	0.044	3.93 (2.05)	0.055		
	% change in variance (Spanish)	-19.61	0.189	**-31.55**	0.001	-14.54	0.256		
	% change in variance (English)	-7.41	0.273	**-11.90**	0.035	6.63	0.408		
Behavior Problems	HS (ref: English)	-0.17 (0.11)	0.131	-0.01 (0.13)	0.928	0.09 (0.12)	0.450	0.11 (0.17)	0.492
	HS * Spanish	-0.08 (0.31)	0.785	-0.39 (0.23)	0.090	-0.33 (0.25)	0.189	-0.54 (0.30)	0.072
	% change in variance (Spanish)	9.12	0.341	-1.33	0.906	**-21.30**	0.011	**-15.17**	0.043
	% change in variance (English)	-1.78	0.823	-4.36	0.621	-5.23	0.454	-8.95	0.175
Social Skills	HS (ref: English)	0.04 (0.05)	0.378	0.01 (0.05)	0.977	**0.14 (0.05)**	0.015	0.06 (0.06)	0.371
	HS * Spanish	-0.26 (0.15)	0.090	0.04 (0.09)	0.640	-0.17 (0.12)	0.160	-0.06 (0.13)	0.633
	% change in variance (Spanish)	-2.63	0.897	-16.80	0.191	-3.07	0.827	0.23	0.987
	% change in variance (English)	0.19	0.976	-8.93	0.334	-3.35	0.633	2.47	0.703
Social Competency	HS (ref: English)	-0.04 (0.05)	0.421	-0.03 (0.04)	0.494	0.01 (0.04)	0.727		
	HS * Spanish	0.11 (0.08)	0.136	0.08 (0.07)	0.262	0.06 (0.08)	0.438		
	% change in variance (Spanish)	-22.02	0.068	-5.79	0.756	-36.92	0.068		
	% change in variance (English)	15.54	0.143	6.13	0.548	3.55	0.681		
